# Role of glioblastoma stem cells in cancer therapeutic resistance: a perspective on antineoplastic agents from natural sources and chemical derivatives

**DOI:** 10.1186/s13287-021-02231-x

**Published:** 2021-03-24

**Authors:** Ana Laura V. Alves, Izabela N. F. Gomes, Adriana C. Carloni, Marcela N. Rosa, Luciane S. da Silva, Adriane F. Evangelista, Rui Manuel Reis, Viviane Aline O. Silva

**Affiliations:** 1 Molecular Oncology Research Center, Barretos Cancer Hospital, Rua Antenor Duarte Villela, 1331, CEP 14784 400, Barretos, São Paulo, Brazil; 2 Life and Health Sciences Research Institute (ICVS), School of Medicine, University of Minho, 4710-057 Braga, Portugal; 3 ICVS/3B’s PT Government Associate Laboratory, 4806-909 Braga, Portugal

**Keywords:** Chemoradioresistance, Clinical trials, Glial stem cell, Initiating cells, Therapeutic strategies, Natural products

## Abstract

Glioblastoma (GBM) is the highest-grade form of glioma, as well as one of the most aggressive types of cancer, exhibiting rapid cellular growth and highly invasive behavior. Despite significant advances in diagnosis and therapy in recent decades, the outcomes for high-grade gliomas (WHO grades III-IV) remain unfavorable, with a median overall survival time of 15–18 months. The concept of cancer stem cells (CSCs) has emerged and provided new insight into GBM resistance and management. CSCs can self-renew and initiate tumor growth and are also responsible for tumor cell heterogeneity and the induction of systemic immunosuppression. The idea that GBM resistance could be dependent on innate differences in the sensitivity of clonogenic glial stem cells (GSCs) to chemotherapeutic drugs/radiation prompted the scientific community to rethink the understanding of GBM growth and therapies directed at eliminating these cells or modulating their stemness. This review aims to describe major intrinsic and extrinsic mechanisms that mediate chemoradioresistant GSCs and therapies based on antineoplastic agents from natural sources, derivatives, and synthetics used alone or in synergistic combination with conventional treatment. We will also address ongoing clinical trials focused on these promising targets. Although the development of effective therapy for GBM remains a major challenge in molecular oncology, GSC knowledge can offer new directions for a promising future.

## Introduction

**Fig. 1:**
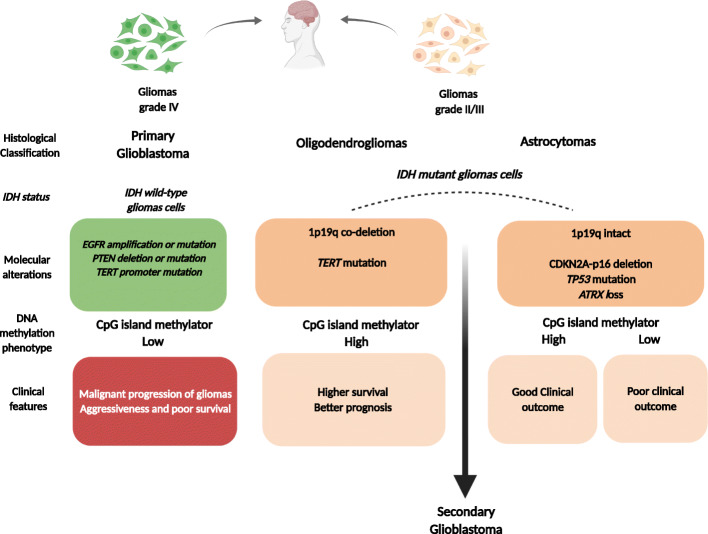
Gliomas classification regarding the mutation status of isocitrate dehydrogenase 1 (IDH-1) gene. See text for details (created with Biorender.com)

Gliomas are the most frequent primary brain tumors in adults, accounting for more than 80% of all malignant cerebral neoplasms [1]. Among these tumors, glioblastoma (GBM) is the most common primary intracranial tumor with a very poor prognosis (WHO grade IV), representing 57.3% of all gliomas [1, 2]. These tumors can be divided into *IDH* wild type, clinically defined as primary or de novo glioblastoma, which corresponds to approximately 90% of GBM cases and generally occurs in patients aged 62 or older, and *IDH* mutant, corresponding to secondary glioblastoma (approximately 10% of cases) that progressively develops from low-grade astrocytoma and frequently manifests in patients aged 40–50 years old (Fig. 1) [2, 3]. Currently, the most frequent molecular alterations associated with primary GBM are epidermal growth factor (*EGFR*) amplification or mutation, loss of heterozygosity (LOH) of chromosome 10q at the phosphatase and tensin homolog (*PTEN*) locus, and *TERT* gene promoter mutation (Fig. 1). Moreover, combined deletion of the complete 1*p* and 19*q* after unbalanced translocation between chromosomes 1 and 19 resulting in the *1p19q* codeletion, homozygous deletion of *CDKN2A*-p16, loss of tumor suppressor genes such as *TP53* and *ATRX*, and *IDH1/2* gene mutations are common molecular alterations found in secondary GBM (Fig. 1) [2, 4]. The amplification of the *EGFR* gene affects the development and progression of gliomas, conferring more aggressive properties, and can be used as a therapeutic target (Fig. 1) [3, 5]. Recent studies showed that the *TERT* promoter mutation essentially accounted for primary GBM and was associated with aggressiveness and poor survival (Fig. 1) [6, 7]. Although the presence of the *1p19q* codeletion is associated with higher survival [8], *CDKN2A-p16* deletion was associated with poor prognosis [8]. The association of *TP53* mutation in GBM and ATRX mutation has not been consistent. So far, it is known that both can co-occur [9]. Importantly, *IDH* mutations are well-established markers of better prognosis [3, 8]. Genomic studies have also described five molecular subclasses (mesenchymal, classical (or proliferative), proneural, neural, and G-CIMP) [10]. Despite improvements in the knowledge and molecular characterization of glioblastomas, no significant difference in patient survival has been observed between primary and secondary glioblastomas, with both showing a mean survival of 12 to 15 months and a high frequency of tumor relapse [11].

The gold standard treatment for GBM patients is surgical resection combined with radiotherapy and adjuvant chemotherapy with the alkylating agent temozolomide (TMZ) [3, 12]. Although some molecular features have been proposed as predictive biomarkers of the treatment response to alkylating agents, such as the methylation status of the O6-methylguanine-DNA-methyltransferase (*MGMT*) promoter, the clinical utility of these markers is minimal [12, 13]. Some reasons proposed for this resistance may include the diffuse infiltrative nature to the surrounding brain, which hinders total resection; the high heterogeneity of GBM, involving distinct molecular pathways; and, more recently, the presence of stem cell-like tumorigenic features, including inducing angiogenesis, uncontrolled cellular proliferation, resisting cell death, and genome instability and mutation [3, 12].

Evidence of small populations of tumor cells that are similar to stem cells, known as cancer stem cells (CSCs) or tumor-initiating cells, has been known as a cause of tumor initiation and development since the nineteenth century and was first described in hematologic malignancies in 1994 [14].

**Fig. 2:**
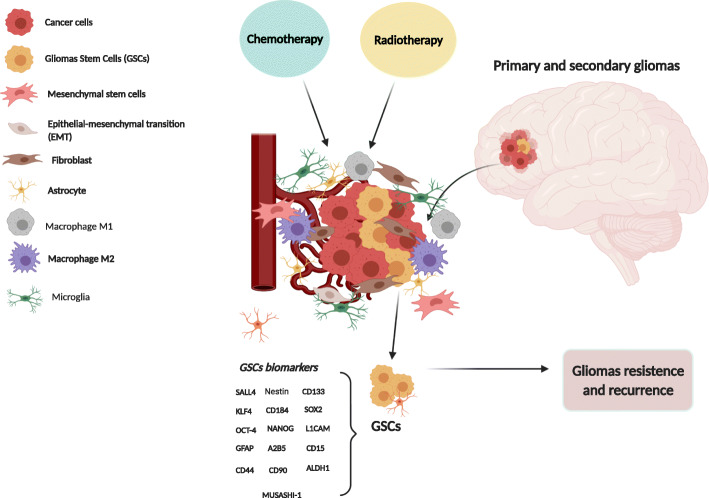
Schematic overview of the cellular components of the microenvironment of glioblastoma (GBM). GSC: glial stem cell; Tumor microenvironment is a complex network composed of stromal cells (fibroblasts, microglia, astrocyte), mesenchymal cells, stem cells, and immune and inflammatory cells (macrophages). The main biomarkers of glial stem cells are indicated (created with Biorender.com)

The first evidence of brain stem cells was shown by Ignatova et al. [15] and later supported by several other groups [16–18]. In glioblastoma, glioblastoma stem cells (GSCs) were first identified by Singh et al. as a population of cells capable of initiating tumor growth in vivo [19]. The first accepted GSC surface marker was CD133 [18]. This marker allows the subdivision of stem cells into two groups: CD133-positive cells (CD133^+^), or cancer stem cells, and CD133-negative cells (CD133^−^), or non-cancer stem cells [20]. CD133 expression also enables the characterization of cell self-renewal capacity, as there is a decrease in the expression of this surface marker during cell differentiation [21]. Another critical feature of CD133^+^ cells is the capacity to generate neurospheres in vitro and induce brain tumor formation in in vivo models [19, 22]. Other markers have also been associated with GSCs that together classify a signature of these cells. According to Dirks and coworkers, CD15 and CD133 are the most useful surface markers of GSCs reported to date and stand out compared to other markers [23]. The presence of dual CD133^+^/Ki-67^+^ cells and associated *Nestin* or *HOX* genes is an adverse prognostic factor for GBM progression [24–26]. Another marker highly expressed in GSCs is the CXCR4 chemokine receptor (CD184), which is associated with CD133^+^ cells and increased expression of hypoxia-inducible factor (*HIF-1-α*) [27, 28]. The same importance should be assigned to the MUSASHI-1 protein, a regulator of translation and cellular fate [29]. Other markers, including the cell-surface glycoprotein CD44; the cell-surface gangliosides A2B5, CD90, and SOX2; and ALDH1, L1CAM, KLF4, SALL4, and GFAP, have also been also used for the identification of GSCs [29–35]. However, the specificity of the surface marker CD133 remains unclear, with groups reporting the identification of GSCs that are CD133 negative [36]. Therefore, it is also noteworthy that although CD133 and CD44 persist on genetically diverse clones [37], the presence of more primitive markers, such as OCT-4, SALL4, and NANOG, among others, needs to be better defined and may be key to developing novel and effective treatments for GBMs [38]. The main biomarkers are summarized in Fig. 2.

Therefore, the promising GSC hypothesis offers new insight into cancer diagnosis and adds complexity in the management of brain tumors. The concept that GBM resistance could be dependent on innate differences in the sensitivity of clonogenic GSCs to chemotherapeutic drugs/radiation stimulated the scientific community to rethink the understanding of GBM growth and therapies designed to be directed at eliminating these cells or modulating their *stemness* [31, 39]. So far, the research strategies involve the development of drugs that target cancer *stemness*, directly or indirectly, in order to target multiple molecules, either alone or in combination.

This review aims to report intrinsic and extrinsic mechanisms that mediate chemoradioresistance in GSCs and therapies based on antitumor agents from natural sources, derivatives, and synthetics used alone or in synergistic combinations with conventional treatments. We will also summarize ongoing clinical trials focused on these promising targets. Although the development of effective therapies for GBM remains a major challenge in molecular oncology, GSC knowledge can offer new directions for a promising future.

## Radioresistance

### Role of repair mechanism in mediating radioresistance

Ionizing radiation (IR) from radiotherapy induces different types of DNA damage, especially DNA double-strand breaks (DSBs). Depending on the type of injury caused, DNA repair mechanisms and DNA damage checkpoints can be triggered, allowing cells to repair DNA damage and proliferate again [40]. The cellular response to DNA damage has been considered as one of the leading survival mechanisms of tumor cells after radiotherapy [40, 41].

Preclinical evidence demonstrates that many of these protection mechanisms are activated in CSC populations, possibly resulting in treatment resistance [42]. Bao et al. showed that CD133^+^ cell subpopulations are resistant to IR due to a more efficient repair system (phosphorylation of CHK1, CHK2, and H2AX form γ-H2AX foci) than the bulk of tumor cells and undergo apoptosis less frequently [31]. Moreover, an increase in CD133^+^ cells was also evidenced in clinical samples from recurrent tumors after high-dose radiotherapy treatment [43, 44]. Among other DNA repair-related genes, *RAD51* overexpression is observed in GSCs, and *BRCA1* and *BRCA2* showed upregulation in glioblastoma cell lines, which led to reduced DNA damage after irradiation [45, 46]. The activation of ataxia-telangiectasia mutated (ATM) and *ATM*- and Rad3-related (*ATR*) also mediates radioresistance [47, 48]. In addition, other authors have reported that, in vitro, CD133^+^ primary cells are more radiosensitive than established glioma cell lines, with a reduced capacity to repair DNA double-strand breaks and an intact G2 checkpoint but no intra-S-phase checkpoint [21]. Therefore, the applicability of CD133^+^ GBM as a model of radioresistance is still unclear. These data highlight the heterogeneity of the in vitro radiosensitivity that exists among primary cell lines and reveal that radioresistance may be independent of the intrinsic GSC characteristics.

### Role of microenvironment in radioresistance

One parameter that may influence radioresponse is the tumor microenvironment. Tumors are comprised of multiple components other than tumor cells (endothelial cells and multiple infiltrating inflammatory and immune cells, together with the extracellular matrix, cytokines, nitric oxide, and oxygen levels) which are also exposed to radiation during therapy, and their crosstalk might influence tumor stem cells’ response to radiation [42, 49]. The critical role of the microenvironment, along with GSCs, is supported directly and indirectly by the observation that GSCs reside in specific niches, distinct compartmentalized regions that present morphologically and functionally distinct functions (Fig. 2) [50]. The stem cell niche plays an indispensable role in homeostasis, regeneration, maintenance, and repair. There are at least three specialized tumor niches in GBM that include the vasculature as an integral regulatory component, including the perivascular tumor niche, vascular-invasive tumor niche, and hypoxic-necrotic tumor niche. These niches are dependent not only on normal cell components in the tumor microenvironment but also on the genetic and epigenetic profiles of GSCs. The different combinations of cell components and functional statuses of the vasculature promote specific features and functions in the niches, as reviewed by Hambardzumyan and Bergers [50]. In addition to GSC maintenance, the niches could undergo dynamic alterations in a temporal and spatial manner and create a succession of tumor microenvironments to accommodate the aggressive growth of a tumor such as GBM into normal tissue, both during tumor progression and in response to therapeutic agents. In this context, some therapies could convert a tumor niche into another niche type instead of eliminating it, thereby losing their effectiveness. According to Hambardzumyan and Bergers, it is likely, for instance, that therapies such as radiation and cytotoxic chemotherapies, which create hypoxia and necrosis, may enhance hypoxic niches that will transition into perivascular tumor niches during tumor relapse [50]. Therefore, the understanding of the crosstalk between GSCs and their niches, which supports GSC self-renewal, tumor invasion, and metastasis, as well as GSC escape from therapy, has become a promising target. In this sense, Mannino and Chalmers proposed that radioresistance is a result of interactions between these cells and microenvironmental factors, i.e., the “microenvironment - stem cell unit” [51]. Brain hypoxia is known to be one of the most critical characteristics in the tumor microenvironment and is associated with the promotion of tumor progression and facilitation of angiogenesis, metabolism, and tumor radioresistance [52, 53], in addition to triggering mechanisms such as hypoxia-inducible factor (HIF) signaling and epithelial-mesenchymal transition (EMT). HIF signaling was also reported to be pivotal in GSC regulation [54]. Low oxygen levels were observed to prevent GSC differentiation, induce neurospheres, and maintain the potential of pluripotent embryonic and *stemness* markers [55, 56]. The link between hypoxic responses and GSCs was suggested by Li and coworkers, who found a differential response of GSCs to the *HIF* family of transcription factors, including promotion of their self-renewal [57]. Likewise, a proof-of-concept study using *HIF* knockdown in GSCs resulted in reduced *stemness* in vitro and in vivo [57]. It has also been described that hypoxia induced the expression of vascular endothelial growth factor (*VEGF*) in *HIF1-* and *HIF2*-dependent GSCs [58]. A close relationship between CD133^+^ cells and vascular structures was also found in a study by Christensen and coworkers [59].

Moreover, two independent groups showed that the CD133^+^ subpopulation is capable of de novo tumor vascularization through direct differentiation into endothelial cells, suggesting that a therapy targeting angiogenic factors would be required to inhibit GBM stem cells and tumor neovascularization [60, 61]. Finally, it has also been shown that GBM cells irradiated under orthotopic conditions have a higher capacity for DSB repair than GBM cells irradiated in vitro, which resulted in the induction of fewer γH2AX and 53BP1 foci in CD133^+^ cells than in CD133^−^ cells [62]. The authors also showed an increase in the percentage of CD133^+^ cells at 7 days after radiation, which persisted at the onset of neurologic symptoms, suggesting that CD133^+^ cells are relatively radioresistant under intracerebral growth conditions [62].

### Role of autophagy in mediating radioresistance

Autophagy is a conserved cellular process that is crucial for maintaining cellular homeostasis and survival and differentiation. Therefore, it is associated with a variety of pathologies [63]. In contrast to apoptosis, autophagy is a double-edged sword that could be either protective or detrimental to cells, depending on the nature of the stimulus (nutrient and growth factor deprivation or an external insult like radiation) and the extent of autophagy induction [64]. Recent studies suggest that autophagy has been recognized as frequently activated in cancer and mediates tumor cells’ response to anticancer therapy, especially radiotherapy, decreasing its efficacy by contributing to GSC maintenance and reducing ROS-associated DNA damage [65, 66]. Moreover, radiation preferentially activates autophagy in CD133^+^ cells and increases the levels of the autophagy-related proteins LC3, ATG5, and ATG12 [67]. The same was found in the radioresistant cell line, in which enhanced autophagic flux and silencing of the *LC3A* gene sensitized mouse xenografts to radiation [68]. However, in a study examining the induction of autophagy by radiation and its role in the radioresistance of GSCs, the authors found that GSCs expressed lower levels of autophagy-related protein LC3 and radiation induced a low degree of autophagy in these cells [69]. Moreover, a recent study showed that autophagy induction by the mammalian targets of rapamycin (mTOR) inhibitor rapamycin triggers GSC differentiation and enhances their radiosensitization in vitro and in vivo, with rapamycin thus becoming a promising tool for radiosensitization in glioma [70, 71].

## Chemoresistance

### Role of repair mechanism mediating chemoresistance

The resistance of GSCs to chemotherapeutic drugs has been well documented, yet the importance of DNA repair remains unclear. A recent study calls into question whether the differential and more efficient DNA repair system is specific to all CSCs, since the effects of TMZ require efficient DNA repair (mismatch repair system) [72, 73]. Moreover, the extensive heterogeneity within GBM can complicate the role of the DNA repair system in GSCs [72]. The DNA repair protein MGMT is the best-characterized repair protein and is a crucial modulator of TMZ chemoresistance in GBM [74–76]. *MGMT* is expressed in GBM at various levels, and reports of its expression in the GSC compartments remain conflicting [77, 78]. Nevertheless, there is consensus that *MGMT* expression substantially increases the resistance of GSC [77–79]. A recent report showed *MGMT* expression in half of the CD133^+^ cell lines tested, and the majority of these cell lines were resistant to TMZ. This result may suggest the presence of an alternative MGMT-independent mechanism of therapeutic resistance [80].

### Role of multidrug mediating chemoresistance

Another mechanism involved in chemoresistance is multidrug resistance. However, its role in GSCs remains an open question. Normal and cancer stem cells have higher expression levels of several ABC transporters, which confer them with efflux ability for the fluorescent dye Hoechst 33342 and helps GBMs with the efflux of antineoplastic drugs [81]. In line with this, increased *ABCG1* expression was reported in TMZ-induced cells (the side population cells in flow cytometry that present the GSC phenotype) [82], and enhanced expression of multidrug resistance 1 (*MDR1*) was found in the chemoresistant phenotype of CD133^+^ GSCs compared to bulk CD133^−^ [83, 84]. Although *ABCB1* can be an independent predictor for TMZ responsiveness [85], there is conflicting data regarding TMZ transport by these proteins. Bleu and coworkers showed that TMZ is not a substrate for the ABCG1 transporter [72, 86] in murine glioma cells.

On the other hand, *ABCG2*/*BCRP* and *ABCB1*/*MDR1* overexpression in GSCs was correlated with higher resistance of GSCs to chemotherapeutic drugs. Accordingly, the use of an ABC transporter inhibitor, such as verapamil, can decrease temozolomide, doxorubicin, and mitoxantrone resistance in GSCs [87]. Besides, melatonin (*N*-acetyl-5-methoxytryptamine) increased methylation levels of the ABC transporter *ABCG2/BCRP* promoter, promoting a synergistic toxic effect with TMZ on GSCs and A172 malignant glioma cells [87]. Moreover, reversan, an inhibitor of the MRP1 protein, increased the sensitivity of primary and recurrent GBM cells to TMZ treatment; however, this effect has not yet been evaluated exclusively in GSCs [88].

### Role of apoptosis and autophagy in mediating chemoresistance

The mechanisms of action of TMZ, such as apoptosis, senescence, and autophagy, have also been described in GSCs [72, 89]. Prolonged treatment with TMZ can induce p53 and p21WAF1/Cip1 and cell cycle arrest (G2/M arrest), although genetic background dependence is observed in GBM cell lines. Continued treatment also promotes apoptosis, but senescence is the major process observed in glioma and melanoma tumors or cell lines [72, 89, 90]. Concerning apoptosis, following TMZ exposure, higher expression levels of antiapoptotic genes were observed in GSCs than in differentiated cell lines, suggesting a possible link between GSC chemoresistance and antiapoptotic factors [84, 91]. It was also reported that drug resistance observed in GSCs might depend on abnormalities in the cell death pathway, such as the overexpression of antiapoptotic factors or silencing of key death effectors [92]. The autophagy process or autophagic cell death induction can also be observed in response to TMZ and contributes to glioma chemoresistance and TMZ treatment failure [93, 94].

Moreover, in GSCs obtained from freshly resected GBM specimens, the expression of autophagy-related proteins (i.e., Beclin-1, ATG55, and LC3) was decreased in CD133^+^ cells compared with CD133^−^ cells after TMZ exposure. The authors suggested that GSCs might not be susceptible to classical pathways of autophagy [95]. On the other hand, rapamycin induces the differentiation of GSCs by activating autophagy [96, 97]. Increased rates and numbers of neurospheres in the rapamycin group compared with other groups were also reported. Additionally, stem/progenitor cell and differentiation markers were downregulated and upregulated in rapamycin-treated cells, respectively [97]. These data suggest that apoptosis and autophagy might contribute to GSC chemoresistance.

### Role of Notch and Sonic hedgehog pathways in mediating chemoresistance

The increased expression of proteins of the Notch and Sonic hedgehog (SHH) pathways in CD133^+^ cells compared with GBM cells has already been described [98]. The comparison between treated and non-treated CD133^+^ primary GBM cells showed upregulated expression of the *NOTCH 1*, *NCOR2*, *HES1*, *HES5*, and *GLI1* genes after TMZ treatment, suggesting the increased activity of these pathways [99]. Moreover, the use of Notch or SHH inhibitors with TMZ reversed the resistance to TMZ [99].

### Epithelial-mesenchymal transition (EMT) mediates GBM chemoresistance

The epithelial-mesenchymal transition process can also contribute to GBM chemoresistance. This was exploited through the gene *ZEB1*, an EMT regulator, and a known regulator of *stemness* and *SOX2* in solid tissue cancers [100]. In GBM, the overexpression of the *ZEB1* gene induced the expression of *MGMT*, resulting in greater tumor chemoresistance and also induced the expression of *SOX2* and *OLIG2*, resulting in greater *stemness* and higher capacity for tumor formation [101].

### Other mechanisms mediating chemoresistance—extrinsic pathways

Previous studies reported that TMZ might eliminate CSCs under in vitro conditions [72, 77]. However, patients treated with TMZ, present no stabilized disease or recurrence, leading to fatal relapses [74], suggesting that other mechanisms, such as residual CSC survival, may occur in vivo. Other extrinsic factors, such as the microenvironment, contribute to the chemoresistance of solid tumors [72]. Hjelmeland and coworkers demonstrated that exposure to an acidic pH environment promoted malignancy in GBM through the induction of a GSC phenotype [102]. Moreover, cell-cell interactions and IL-6 protein expression constitute indirect evidence suggesting that these mechanisms may be relevant for GSCs [72, 103]. Several reports suggest that tumor cell *stemness* could be induced by tumor microenvironments such as hypoxia [57, 79, 104] and drugs such as TMZ [80]. In line with this, tumor cells can acquire CSC properties [80, 105]. A recent report showed essential data concerning the origin, development, and maintenance of the GSC population after TMZ treatment. In this study, the authors achieve the conversion of non-GSCs to GSCs, both in vitro and in vivo, after long-term exposure to clinically relevant doses of TMZ. They showed that newly formed GSCs expressed molecular markers associated with parental GSCs, displayed a high rate of tumor engraftment and had a more invasive phenotype. These data suggest that the *stemness* of GSCs may be governed by cellular plasticity and that TMZ can stimulate the dedifferentiation of non-GSCs, explaining the high rates of tumor recurrence after conventional therapy [80].

## Current strategies targeting cancer stem cells

**Fig. 3:**
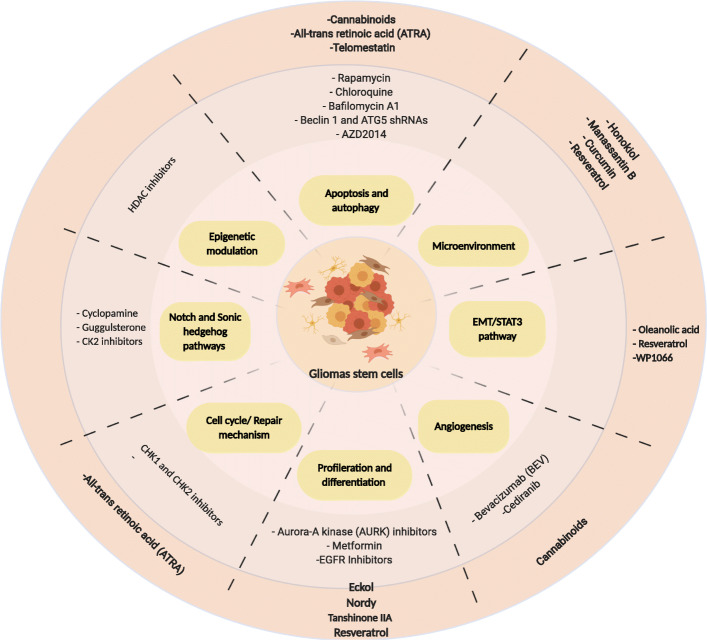
A schematic representation of the molecular signaling hallmarks of glial stem cell (CSC) and the effect of natural compounds and synthetic drugs on these molecular targets. In the dark red circle are represented natural compounds that target each hallmark. In light red are represented chemicals/synthetic drugs that targeted each hallmark in GSC. See text for details (created with Biorender.com)

The discovery of pathways essential for modulating *stemness* properties has contributed to the identification of several molecules that could eliminate GSCs, including new antineoplastic agents from natural sources [106–113]. A summary of current potential treatments is presented in Table 1 and Fig. 3.

Given the requirement for hedgehog (Hh) signaling in GSCs, a recent study investigated cyclopamine (11-deoxojervine) (1) and guggulsterone (2) [114, 185]. Cyclopamine can specifically inhibit the Hh pathway [117] as well as the side and aldefluor-positive populations, resulting in cultures unable to form colonies in preclinical studies and GSCs sensitized to radiation [118, 185]. Another important protein able to target different pathways (*Hedgehog*, *Notch*, and *β-catenin*) is casein kinase 2 (CK2) [119]. In GBM, *CK2* expression and activity lead to tumor suppressor inhibition and oncogene activation contributing to gliomagenesis [119]. Moreover, *CK2* inhibition promotes O6-methylguanine-DNA-methyltransferase downregulation and sensitizes glioma cells to TMZ [121]. CX-4945 (3) is potent, selective, and highly bioavailable compared to other CK2 inhibitors [121]. This novel molecule and its analogs target several GSC factors and markers and exhibit promising results in preclinical studies of several types of tumors, in vivo models and human clinical trials [121, 186].

Regarding the cell cycle and DNA damage repair [122, 123], there are several CHK1 and CHK2 inhibitors; CHK1 inhibitors in clinical development include SCH 900776 (NCT00779584) (4) and SAR-020106 (5) [124, 125]. AZD7762 (NCT00413686) (6) and debromohymenialdisine (DBH) (7) are novel potent checkpoint kinase inhibitors that inhibit both *CHK1* and *CHK2*. AZD7762 was shown to potentiate chemotherapy response in several different settings and resulted in the abrogation of DNA damage-induced cell cycle arrest in vitro and in vivo in combination with DNA-damaging agents [126, 127]. The use of DBH and radiation treatment is synergistic: together they are able to abrogate the radioresistance of CD133^+^ cells, suggesting new options for combination radiotherapy [31, 128].

EGFR is a crucial receptor in the protocols described for growing GSCs, making clear that this pathway is necessary for GSC survival [129, 130]. Thus, it would be rational to use *EGFR* inhibitors to promote the inhibition of GSC proliferation and self-renewal and induce cell death [129, 137]. First-generation *EGFR* inhibitors such as erlotinib (8) and gefitinib (9) have been used in the clinical treatment of glioma patients, although less than 20% of patients presented a response to these treatments. However, *EGFR* inhibition has been observed to enhance the chemo- and radiosensitivity of human glioma CSCs [136, 137]. It is believed that the low response to these inhibitors is associated with loss of the tumor suppressor *PTEN*, which is usually deleted or mutated in gliomas and plays a critical role in maintaining neural precursor cells via activation of the mTOR pathway [133, 139]. Clinical results with rapamycin (10) have been described in patients with high-grade GBM [140, 141] and have demonstrated that treatment with rapamycin or combination with EGFR inhibitors may provide an alternative treatment for TMZ-resistant gliomas, regardless of *EGFR* status [134, 142].

Related to autophagy, the cell death and survival response can be influenced to favor cell death through several therapies that inhibit autophagy processes [155]. Chloroquine (CQ) (11) is an applicable autophagy inhibitor known to trigger apoptosis in conventional autophagic tumor cells and to improve mid-term survival in glioma when administered in addition to conventional therapy [187, 188]. Regarding GSCs, triple combinations of γIR, low-dose CQ, and PI3K/Akt pathway inhibitors or high-dose CQ alone induced strong cytotoxic effects in radioresistant GSCs [187]. Moreover, inhibitors such as bafilomycin A1 or beclin 1 and ATG5 shRNAs also sensitize GSCs to radiation and reduce their viability and capacity to form neurospheres [134, 189]. Likewise, radiation and the inhibition of αv integrin by cilengitide (12), which is currently in clinical evaluation, induce autophagy in GSCs, increasing cytotoxicity and reducing cell survival [190].

In addition, the association of mTOR inhibitors and radiation led authors to evaluate the effects of AZD2014 (13) [156], a competitive dual mTORC1/2 inhibitor, unlike rapamycin, an allosteric inhibitor, on the radiosensitivity of GSCs in in vitro and in vivo studies [191]. Beyond these properties, AZD2014 also penetrates the blood-brain barrier and has been reported in a Phase I clinical trial as a single agent [156, 159, 160]. The authors showed that AZD2014-mediated radiosensitization in GSCs promoted the inhibition of DSB repair as evaluated by a clonogenic assay according to γH2AX foci. Additionally, in GSC-initiated orthotopic xenografts, AZD2014, when combined with radiation, significantly prolonged mouse survival even when administered for only 3 days. These data indicate that AZD2014 may be a radiosensitizer applicable to GBM therapy [192].

Moreover, other molecules can decrease the *stemness* properties of GSCs, including eckol (14) [107], Nordy (15) [108], resveratrol (16) [109], STX-0119 (17) [161], ER400583-00 (18) [110], WP1193 (19) [111], angiogenesis inhibitors [143], all-*trans*-retinoic acid (20) [144], and Tanshinone IIA (21) [145]. Some of these molecules are linked to targets the microenvironment and thus indirectly modulate the *stemness* properties of cancer cells. However, these drugs cannot be used for a specific tumor type because the role of each niche in different tumor types and how they differ from one another is not yet known. This class includes molecules that primarily target angiogenesis and hypoxia [107, 108, 110, 111, 145].

Eckol (14), a phlorotannin compound from *Ecklonia* species, has been shown to attenuate in vitro anchorage-independent growth on soft agar and reduce sphere formation and GSC markers. Moreover, the CD133^+^ subpopulation and self-renewal-related proteins were decreased in response. The authors suggested that eckol activity could target PI3K/Akt and MAPK signaling pathways. Importantly, eckol treatment also decreases the resistance of GSCs to IR and TMZ and tumor formation in xenograft mice [107, 146].

The synthetic dl-nordihydroguaiaretic acid compound Nordy (15) was also shown to inhibit self-renewal properties, induce GSC differentiation, and decrease the GSC pool in vitro and in vivo. Alox-5 is a Nordy target that promotes the invasion and proliferation inhibition of GSCs. Moreover, Nordy promotes *GFAP* upregulation, angiogenesis inhibition, and *stemness* marker downregulation [108, 147].

Regarding pluripotency, signal transducer and activator of transcription 3 (*STAT3*), which is associated with the cell cycle and survival, regulation, immune response, and differentiation, has been described as a critical initiator and regulator of tumorigenic transformation in GBM and implicated among GSC maintenance factors. STAT3 has been related to oncogenic or tumor-suppressive roles in GBM depending on the tumor genotype [111, 167]. These novel therapies may be the basis for the next generation of GBM treatment. STAT3 signaling includes small molecules such as oleanolic acid (22) [168], STX-0119 (13) [161], and WP1066 (23) [157]. Resveratrol (16) (RV), a polyphenol in grapes, is known to be a potential noncytotoxic tumor-preventive drug targeting STAT3 signaling. In glioma, RV can induce apoptosis, enhance radiosensitivity in the CD133^+^ cell population, and decrease tumorigenicity in xenotransplant experiments. Furthermore, RV was able to inhibit cell proliferation and decrease cell motility by modulating the Wnt signaling pathway and EMT activators [109, 193].

Another novel molecule recently investigated is WP1066 (23), an analog of the natural product caffeic acid benzyl ester and a potent STAT3 pathway inhibitor. In glioma, this potent small-molecule inhibitor showed promise as a therapeutic agent by targeting GSCs and will be investigated in a clinical trial for patients with recurrent malignant glioma and brain metastasis from melanoma (recruiting, ClinicalTrials.gov). In addition, WP1066 can cross the blood-brain barrier and is orally bioavailable [157, 194].

Antiangiogenic agents that disrupt GBM-initiating cell maintenance have been widely investigated, but so far, only modest results have been obtained. Moreover, some reports have indicated that glioma develops resistance to the employed antiangiogenic treatments [195, 196]. To date, in highly vascular tumors such as gliomas [149, 197], angiogenesis inhibition has improved progression-free survival, although no cure has been achieved. Clinical trials using bevacizumab (BEV) (24) and cediranib (AZD2171) (25) (Phase I) alone or in combination have demonstrated efficacy in GBM patients [164, 165]. In in vivo experimental studies with mice, BEV treatment decreased GSCs and the growth rate of GBMs [2].

Regarding clinical trials, BEV (24) has been combined with irinotecan (Phase II) and pazopanib, also an oral multitarget angiogenesis inhibitor (GW786034) (Phases I and II) [143, 164, 165]. A study also demonstrated that BEV or interferon-beta could enhance radiosensitivity in orthotopic GBM [171]. However, recent studies suggest that inhibition of angiogenesis is even a driving force for tumor conversion to a higher malignancy state, inducing a phenotypic change from single-cell infiltration to migration of cell clusters along normal blood vessels, which is reflected in higher invasion, enhanced metastatic activity and dissemination [150, 196].

Moreover, antiangiogenic therapy changes tumor vasculature, leading to hypoxia [196]. The hypoxia phenotype has been demonstrated as a marker of antiangiogenic therapy resistance by *HIF-1α* and stromal-cell derived factor-1α (*SDF-1α*) upregulation leading to the recruitment of various pro-angiogenic bone marrow-derived cells [196]. For hypoxia modulation, compelling data demonstrate that the downregulation of *HIF2*-α can increase stem cell/pluripotency markers, neurosphere formation, and the VEGF pathway [57, 151]. Thus, several molecules that inhibit or indirectly modulate the expression of *HIF-1* have been investigated, although they showed little efficacy either alone or in combination with standard antitumor agents [152]. For instance, honokiol (26), manassantin (27) B from *Saururus cernuus* and *Saururus chinensis*, curcumin from *Curcuma* spp. (28), resveratrol (16), SU5416 (29), and ER400583-00 (18) are inhibitors that have been developed [106, 109, 110, 153, 164, 198, 199].

Honokiol (26) is one of the biphenolic bioactive compounds isolated from *Magnolia officinalis*, possessing multifunctional activities in addition to crossing the blood-brain barrier [173, 174]. Honokiol specifically inhibits PI3K/mTOR signaling activation in gliomas [173, 175], promotes the elimination of GSCs, and reverses TMZ resistance using GBM8401 SP cells, which appear to have higher expression of *MGMT* and to be more resistant to TMZ. This inhibition is accompanied by a greater induction of apoptosis and reduced expression levels of *EGFR*, CD133, and *Nestin*, suggesting that honokiol might have clinical benefits for GBM patients, mainly those who are refractory to TMZ treatment [75, 173, 176].

Furthermore, several agents, such as cannabinoids (30), are known to exert antitumor action on GBM by apoptosis induction and tumor angiogenesis inhibition and have been recently evaluated in GSC differentiation control. The study provides further support for the hypothesis that cannabinoid changes reduce glioma initiation (neurosphere formation and cell proliferation) in vivo. These effects were greater in combination with TMZ and correlated with an increase in cell differentiation [177, 200]. Another strategy to reduce the tumorigenic potential of GSCs and promote differentiation is to induce bone morphogenic proteins (BMP) signaling [165, 178]. The potential of *BMP* induction of astrocyte differentiation from normal neural precursors has been reported in vitro and in vivo [149, 205]. Most importantly, *BMP4* has been shown to trigger a significant decrease in GSCs [165, 178, 179]. Even with BMP signaling, it was identified that *BMP2* heightens sensitivity to TMZ in GSCs in which *MGMT* expression was described as directly downregulated by *HIF-1*α at the transcriptional level [180, 201].

The epigenetic modulation of histone acetylation by histone deacetylase (*HDAC*) activity has been associated with several cancer types [182, 202]. The HDAC inhibitor, vorinostat (suberoylanilide hydroxamic acid, SAHA) (31), and sahaquine (32) are currently in Phase I clinical trials [138, 183, 203] in GBM. In vitro, SAHA has shown antiproliferative effects by blocking G1/S phase progression, increasing the levels of apoptosis-related genes and inducing the expression of cleaved PARP and p-γH2AX in GSCs [204]. Sahaquine inhibits *HDAC6,* leading to a reduction in the viability and invasiveness of glioblastoma tumors and brain tumor stem cells [138]. Most importantly, HDAC inhibitor treatment under culture proliferation conditions suggests the induction of differentiated cell states in adult mouse neural stem cells [205].

In addition, *all-trans-*retinoic acid (ATRA) (20), a differentiating agent used in clinical practice, is a natural compound derivative of retinoic acid, also known as vitamin A [202, 206]. Some findings reported its capacity for differentiating stem cells as well as normal neural progenitor cells and downregulating the expression of the stem cell marker nestin [207, 208]. An additional study revealed that a combination of ATRA and paclitaxel was able to synergistically reduce GBM tumor growth in both in vivo and in vitro models [209]. GSCs differentiated into glial and neuronal lineages even at low concentrations of ATRA. Moreover, ATRA decreased proliferation and self-renewal of neurospheres and promoted apoptosis at high concentrations, targeted ERK1/2 signaling, induced cell cycle arrest at the G1/G0 to S transition, decreased cyclin D1 expression, and increased p27 expression [206, 210].

Another agent with antiproliferative and prodifferentiation effects in GSCs is aurora-A kinase (AURK), a crucial serine-threonine kinase [211, 212] observed to be variably overexpressed in gliomas [211, 213]. AURK is also a vital kinase that governs self-renewal capacity in GBM tumorsphere cultures [172]. These authors used a pan-AURK inhibitor, VX680 (33), followed by radiation, in cell culture and xenograft models, and demonstrated the induction of apoptosis and reduction of tumor growth [172]. Hong and coworkers also demonstrated that MLN8237 (alisertib) (34) [214–216], a highly selective AURK inhibitor, inhibits colony formation in GSCs and potentiates the effects of radiation and TMZ in glioblastoma monolayers and GSCs [217]. Importantly, MLN8237 is relatively non-toxic to normal human astrocytes.

Another emerging antiglioma drug is metformin (35), a drug used mainly for the therapy of type 2 diabetes and polycystic ovary syndrome [218, 219]. Metformin treatment in vitro was able to block cell cycle progression (G0/G1 phase), although cell death was not observed in GBMs [220]. In a recent report, metformin selectively and remarkably affects GSC viability in vitro [221]. The authors showed that *AKT* and the transcription factor forkhead box O3 (*FOXO3*) are involved in the molecular mechanism of metformin activity in GSCs [222, 223]. The effects of metformin were also validated in preclinical glioma orthotopic animal models, in which metformin administration resulted in a decrease of the self-renewing properties and tumor-initiating subpopulation [162, 224]. Clinical trials on the use of metformin alone and cancer treatment (including glioma) and prevention are ongoing [219, 224].

Maruccia and coworkers reviewed exciting results obtained with forty-nine different natural products, including flavonoids, alkaloids, polyketides, and acid derivatives [225]. One terpene or terpenoid compound class that has been reported in GSCs is the retinoic acids (*all-trans*), which are potent differentiating agents [144, 226]. The retinoic acids induce the in vitro differentiation of GSCs and impair the secretion of angiogenic cytokines and GSC motility, promoting synergistic therapy. The antitumor mechanism is associated with the downregulation of Wnt/-catenin signaling [166]. Well-characterized polyketides are telomestatin (36) and derivatives from S*treptomyces anulatus*, able to induce apoptosis and impair migration potential of GSCs in vitro and in vivo and to moderately change non-GSCs and normal neural precursors. Moreover, these macrocyclic compounds also promoted telomeric and nontelomeric DNA damage in GSCs [227, 228]. Two different alkaloids have been reported to be active against GSCs: cyclopamine (1) from *Veratrum californicum* and harmine (37) from *Peganum harmala* [55, 115, 170]. It was demonstrated that harmine inhibits self-renewal and induces GSC differentiation. In particular, harmine inhibits neurosphere formation of human primary glioblastoma GSCs and AKT phosphorylation [229].

**Table 1:** Summary of current therapeutic strategies for some natural products and their chemical derivatives in GSCs. The structure, biological targets, analysis methods, clinical phase andchanism of 38 natural compounds/or derivatives are described

Compound	Therapeutic/structure	Biological targets/mechanism of action	Evaluated	Clinical trials	Ref.
**1**	Cyclopamine (11-deoxojervine)	Target Hedgehog pathway. Promote inhibition of side and aldefluor-positive populations.	In vitro and in vivo	No	[114–116] PubChem CID: 442972
**2**	Guggulsterone	Promote intrinsic apoptosis of GSCs and sensitize cells to SANT-1. Targeting Ros/NF-**κ**B and Hedgehog.	In vitro	No	[115, 117] PubChem CID: 6450278
**3**	CX-4945 (Silmitasertib)	Target several GSC factors and markers Casein kinase 2 selective inhibitor.	In vitro and in vivo	Yes	[118–121] PubChem CID: 24748573
**4**	SCH 900776	DNA repair inhibitors Promote radio-chemosensitivity. Promote CHK1 inhibition.	In vitro and in vivo	Yes	[122–125] PubChem CID: 46239015
**5**	SAR-020106	DNA repair inhibitor Promote CHK1 inhibition	In vitro and in vivo	Yes	[124, 125] PubChem CID: 44203948
**6**	AZD7762	DNA repair inhibitor Promote radio-chemosensitivity. Promote CHK1, CHK2, and ATM protein inhibition.	In vitro and in vivo	Yes	[120, 124] PubChem CID: 11152667
**7**	Debromohymenialdisine (DBH)	DNA inhibitors Promote radio-chemosensitivity. Promote CHK1, CHK2, and ATM protein inhibition.	In vitro and in vivo	Yes	[43, 126–128] PubChem CID: 135451156
**8**	Erlotinib	EGFR inhibitors Promote proliferation and self-renewal inhibition. Promote cell death.	In vitro and in vivo	Yes	[126–132] PubChem CID: 176870
**9**	Gefitinib	EGFR inhibitors Promote proliferation and self-renewal inhibition. Promote cell death.	In vitro and in vivo	Yes	[126–132] PubChem CID: 123631
**10**	Rapamycin (Sirolimus)	Promote GSC differentiation. Reduce stem cell markers. Promote radiosensitivity.	In vitro and in vivo	Yes	[36, 67, 69, 97, 130, 133–135] PubChem CID: 5284616
**11**	Chloroquine (CQ)	Promote radiosensitivity Inhibit autophagy process.	In vitro and in vivo	Yes	[124, 136, 137] PubChem CID: 2719
**12**	Cilengitide	Promote αv integrin inhibition. Promote GSCs autophagy, cytotoxicity, and cell death Promote radiosensitivity.	In vitro and in vivo	Yes	[138] PubChem CID: 176873
**13**	AZD2014 (Vistusertib)	Promote mTORC1/2 inhibition. Promote radiosensitivity. Promote DNA double-strand break repair inhibition.	In vitro and in vivo	Yes	[133, 139–141] PubChem CID: 25262792
**14**	Eckol	Promote radiosensitivity and TMZ sensitivity. Reduce neurosphere formation and stem cell markers Target PI3-kinase-Akt and Ras-Raf-1-Erk signaling pathways.	In vitro and in vivo	No	[107] PubChem CID: 145937
**15**	Nordy	Promote GSC differentiation. Reduce proliferation, stem cell markers, and self-renewal Target ALOX5	In vitro and in vivo	No	[108] PubChem CID: 319062914
**16**	Resveratrol	Promote radiosensitivity and differentiation of GSCs. HIF inhibitor. Induce apoptosis of CD133^+^ cells. Target STAT3 pathway	In vitro and in vivo	No	[106, 109] PubChem CID: 445154
**17**	STX-0119	Promote inhibition of GSCs proliferation. Reduce stem cell markers. STAT3 inhibitor.	In vitro and in vivo	Yes	[142] PubChem CID: 4253236
**18**	ER400583-00	HIF inhibitors Reduce neurosphere formation and stem cell markers Inhibit VEGF signaling Promote microenvironment modulation. Reduce HIF-1 expression.	In vitro and in vivo	No	[57, 106, 111, 143–148] Not available on PubChem
**19**	WP1193	Analog of natural product caffeic acid benzyl ester Reduce proliferation and stem cell markers Induce apoptosis Promote G1 arrest decrease of cyclin D1 and p21(Cip1/Waf-1) increase Inhibit JAK2/STAT3	In vitro and in vivo	No	[112] Not available on PubChem
**20**	All-*trans*-retinoic acid (Vitamin A acid)	Promote differentiation Reduce proliferation and nestin stem cell markers Promote apoptosis Target ERK1/2 signaling	In vitro and in vivo	No	[149–154] PubChem CID: 444795
**21**	Tanshinone IIA	Promote suppression of GSC proliferation. Reduce stem cell markers. Promotes the increase of GSCs differentiation markers. Induce GSCs apoptosis. Reduce the IL6/STAT3 signaling pathway.	In vitro and in vivo	No	[155] PubChem CID: 164676
**22**	Oleanolic acid	Promote suppression of JAK-STAT3 activation in M2 polarization of tumor-associated macrophages.	In vitro	No	[156] PubChem CID: 10494
**23**	WP1066	Promote STAT3 inhibition Decrease the surviving fraction of GSC	In vitro and in vivo	Yes	[157, 158] Not available on PubChem
**24**	Bevacizumab >Bevacizumab light chain DIQMTQSPSSLSASVGDRVTITCSASQDISNYLNWYQQKPGKAPKVLIYFTSSLHSGVPSRFSGSGSGTDFTLTISSLQPEDFATYYCQQYSTVPWTFGQGTKVEIKRTVAAPSVFIFPPSDEQLKSGTASVVCLLNNFYPREAKVQWKVDNALQSGNSQESVTEQDSKDSTYSLSSTLTLSKADYEKHKVYACEVTHQGLSSPVTKSFNRGEC >Bevacizumab heavy chain EVQLVESGGGLVQPGGSLRLSCAASGYTFTNYGMNWVRQAPGKGLEWVGWINTYTGEPTYAADFKRRFTFSLDTSKSTAYLQMNSLRAEDTAVYYCAKYPHYYGSSHWYFDVWGQGTLVTVSSASTKGPSVFPLAPSSKSTSGGTAALGCLVKDYFPEPVTVSWNSGALTSGVHTFPAVLQSSGLYSLSSVVTVPSSSLGTQTYICNVNHKPSNTKVDKKVEPKSCDKTHTCPPCPAPELLGGPSVFLFPPKPKDTLMISRTPEVTCVVVDVSHEDPEVKFNWYVDGVEVHNAKTKPREEQYNSTYRVVSVLTVLHQDWLNGKEYKCKVSNKALPAPIEKTISKAKGQPREPQVYTLPPSREEMTKNQVSLTCLVKGFYPSDIAVEWESNGQPENNYKTTPPVLDSDGSFFLYSKLTVDKSRWQQGNVFSCSVMHEALHNHYTQKSLSLSPGK	Promote disruption of vascular niche and reduce tumor proliferation. Promote radiosensitivity. Reduce proliferation and block GSC ability to induce endothelial cell migration. VEGF inhibitor	In vitro and in vivo	Yes	[113, 124, 132, 159–163] DrugBank: DB00112
**25**	Cediranib (AZD2171)	Promote disruption of the vascular niche and reduce tumor proliferation. Promote radiosensitivity. Reduce proliferation and block GSC ability to induce endothelial cell migration. VEGF inhibitor	In vitro and in vivo	Yes	[164–166] PubChem CID: 9933475
**26**	Honokiol	Promote PI3K/mTOR signaling inhibition. Promote proliferation inhibition of side positive populations. Promote TMZ-resistant cell sensitivity. Promote DNA double-strand break repair inhibition.	In vitro and in vivo	Yes	[75, 95, 167–169] PubChem CID: 72303
**27**	Manassantin B	Target hypoxia-inducible factor-1.	In vitro	No	[111] PubChem CID: 10439828
**28**	Curcumin	Induce GSCs apoptosis. Target hypoxia-inducible factor-1.	In vitro and in vivo	No	[145, 148, 170] PubChem CID: 969516
**29**	SU5416 (Semaxinib)	Reduce neurosphere formation and stem cell markers Reduce HIF-1 expression Inhibit VEGF signaling Target PI3K/AKT/p70S6K1 signaling pathway	In vitro and in vivo	No	[147] PubChem CID: 5329098
**30**	Cannabinoids therapies	Promote differentiation. Inhibit gliomagenesis. Target cannabinoid type 1 (CB1) and type 2 (CB2) receptors.	In vitro and in vivo	No	[157] PubChem CID: 9852188
**31**	Vorinostat (SAHA)	Promote GSC differentiation. Promote G1/S arrest of GSCs. Inhibit histone deacetylases (HDACs).	In vitro and in vivo	Yes	[150, 164, 165, 171] PubChem CID: 5311
**32**	Sahaquine	Promote HDAC inhibition Reduce GSC viability Reduce invasiveness	In vitro	No	[138] Not available on PubChem
**33**	VX680	Pan-AURK inhibitor Induce apoptosis Reduce tumor growth	In vitro and in vivo	No	[172] PubChem CID 5494449
**34**	MLN8237 (Alisertib)	Promote GSC colony formation inhibition. Promote radiosensitivity and TMZ sensitivity. Aurora-A kinase inhibitor.	In vitro and in vivo	No	[173–175] PubChem CID: 24771867
**35**	Metformin	Promote inhibition of GSC self-renewal. Reduce GSC viability. Promote inhibition of CD133^+^ proliferation.	In vitro and in vivo	No	[176–181] PubChem CID: 4091
**36**	Telomestatin	Polyketide component Induce apoptosis and impair the migration potential of GSCs. Promote telomeric and nontelomeric DNA damage in GSCs.	In vitro and in vivo	No	[182] PubChem CID: 443590
**37**	Harmine	Alkaloid component Promote self-renewal inhibition. Promote GSC differentiation. Promote neurosphere formation inhibition.	In vitro and in vivo	No	[183] PubChem CID: 5280953

* For chemical structures, SDF files were retrieved from PubChem [184], and 2D structures were built on MarvinSketch (MarvinSketch 19.27.0, 2019, ChemAxon (http://www.chemaxon.com)

## Conclusion

The development of effective therapy for GBM remains a significant challenge in molecular oncology due to several questions mentioned above. Here, we have discussed several bioactive products that have been reported to modulate GSCs and shown to be essential for therapeutic applications. Although the detailed underlying mechanisms are unknown, bioactive products hold promise for the development of new drugs to treat glioma. Advances in understanding the pathomechanisms of glioma and the identification of GSC properties and therapeutic targets in the GSC subpopulation offer new directions for the development of novel therapies, either isolated or in combination, using personalized targeting for primary brain tumors, which is further emphasized in strategies for basic and translational research with natural compounds.

Papers of special note have been highlighted as either of interest (76, 93) or of considerable interest (15, 39, 78, 112) to readers

## Data Availability

Not applicable.

## Abbreviations

ATM: Ataxia-telangiectasia mutated
ATRA: All-trans retinoic acid
ATR: Ataxia-telangiectasia and Rad3 related
ATRX: Alpha-thalassemia/mental retardation syndrome X-linked
BMP: Bone morphogenic proteins
Bev: Bevacizumab
CSC: Cancer stem cell
CXCR4: C-X-C motif chemokine receptor 4
CDKN2A: Cyclin-dependent kinase inhibitor 2A
CD184: CXCR4 chemokine receptor
CQ: Chloroquine
DBH: Debromohymenialdisine
DSBs: DNA double-strand breaks
FOXO3: Forkhead box O3
GBM: Glioblastoma
GFAP: Glial fibrillary acidic protein
GSC: Glial stem cell
EMT: Epithelial-mesenchymal transition
HDACs: Histone deacetylase
HIF: Hypoxia-inducible factor
IDH-1: Isocitrate dehydrogenase 1
TP53: Tumor protein P53
IR: Ionizing radiation
EFGR: Epidermal growth factor receptor
LC3: Microtubule-associated protein 1A/1B-light chain 3
MGMT: O6-methylguanine-DNA-methyltransferase
MDR1: Expression of multidrug resistance 1
mTOR: Mammalian targets of rapamycin
PTEN: Phosphatase and tensin homolog
TERT: Telomerase reverse transcriptase
Rv: Resveratrol
STAT3: Activator of transcription 3
SDF1 α: Stromal-cell derived factor-1α
TMZ: Temozolomide
VEGF: Vascular endothelial growth factor
